# Salivary Metabolic Characteristics and Response to Neoadjuvant Systemic Therapy in Breast Cancer

**DOI:** 10.3390/ijms27104472

**Published:** 2026-05-16

**Authors:** Lyudmila V. Bel’skaya

**Affiliations:** Biochemistry Research Laboratory, Omsk State Pedagogical University, 644099 Omsk, Russia; belskaya@omgpu.ru

**Keywords:** breast cancer, neoadjuvant chemotherapy, pathological response, pathomorphism, saliva, treatment response, prognostic biomarkers

## Abstract

Metabolic changes in saliva are known to be closely associated with the presence of non-oral cancers, particularly breast cancer. The diagnostic and prognostic potential of salivary biomarkers in breast cancer has been demonstrated, but their applicability for assessing therapy response has not yet been established. The aim of this study was to comprehensively analyze clinical, pathological, molecular, and salivary characteristics when assessing the response to neoadjuvant chemotherapy for breast cancer. The study included 361 breast cancer patients undergoing their first course of chemotherapy and 127 healthy volunteers without breast pathologies. Saliva samples were collected from all volunteers before treatment. Saliva analysis results for amino acids, lipids, and tumor markers were compared with tumor pathomorphism assessment after breast cancer surgery. The proportion of patients with a complete response to therapy was statistically significantly lower after menopause, and in those with HER2-negative breast cancer, moderate tumor differentiation, and high estrogen and progesterone receptor expression. For the first time, a body mass index (BMI) greater than 25 and low HER2 expression (HER2-low) were shown to have an unfavorable prognosis. The criterion for selecting informative salivary metabolites was a multidirectional change in minimal and complete pathological responses to therapy compared to healthy controls. Thus, prognostically favorable signs were a decrease in the concentration of urea below 7.5 mmol/L (OR = 1.921; 95% CI 1.061–4.270; *p* = 0.0342), a decrease in the area of the absorption band at 2957 cm^−1^ below 24 (OR = 3.875; 95% CI 1.160–12.70; *p* = 0.0003), and an increase in the concentration of cancer antigen CA27.29 above 3 U/L (OR = 2.138; 95% CI 1.021–7.273; *p* = 0.0343) and CA-15-3 above 39 U/L (OR = 3.896; 95% CI 1.062–14.07; *p* = 0.0072). With a simultaneous increase in both CA27.29 and CA15-3, the probability of a complete response to therapy increased (OR = 4.288; 95% CI 1.056–17.09; *p* = 0.0013). Multivariate analysis showed that an independent prognostic indicator, along with the expression status of HER2, estrogen receptors, differentiation degree, BMI, and menopause status, was the concentration of CA15-3 in saliva (AUC = 0.789, 95% CI: 0.737–0.842, *p* = 0.0001). Identifying new markers will help physicians formulate treatment plans tailored to a patient’s individual risk factors, leading to increased survival and improved quality of life.

## 1. Introduction

A global trend in the treatment of aggressive stage II–III breast cancer is neoadjuvant chemotherapy (NACT) [1,2]. This initial drug-based approach improves immediate patient outcomes, including reducing locoregional treatment time. Additional stratification of patients into a favorable prognosis group (those who have achieved complete tumor regression at the neoadjuvant stage) and a high-risk group (those with residual tumor) allows for improved survival through escalation of subsequent adjuvant therapy [3,4]. It is also necessary to evaluate the pathological response of the tumor to NACT, which is a strategically important factor for developing a further treatment algorithm and assessing the prognosis for breast cancer [5,6].

Numerous attempts have been made to find prognostic biomarkers of response to NACT, but there is currently no definitive solution [7]. A number of studies have shown that neutrophil-to-lymphocyte ratios (NLRs), platelet-to-lymphocyte ratios (PLRs) [8], lymphocyte-to-monocyte ratios (LMRs), the systemic inflammatory response index (SIRI) [9], tissue biomarkers sTILs [10,11,12], mutational spectrum [13], cfDNA, mtDNA [14], and plasma microRNA [15] can be used for these purposes.

There is extensive evidence for the clinical efficacy of the 21-gene assay (Oncotype DX) and 70-gene assay (MammaPrint) for predicting breast cancer recurrence and chemotherapy response [16]. Gene signatures have been proposed for predicting pathological complete response [17], and comprehensive CDx testing has been proposed for predicting response to NACT and disease progression in patients with triple-negative breast cancer (TNBC) [18]. HMGB1 protein has been identified as a potential marker using quantitative proteomics and immunohistochemistry [19]. RANK expression served as an independent predictor of response to NACT in patients with the luminal-like HER2-negative subtype [20]. The MammaTyper^®^ binary score has shown good results in predicting complete remission in cases of HR+/HER2-negative cancer [21]. Diffuse optical spectroscopy can help identify patients who require additional molecular genetic testing of the tumor to find the source of treatment resistance and adjust the treatment regimen [22]. Metabolomic approaches that allow dividing patients into groups with low and high risk of relapse regardless of classical prognostic factors have yielded promising results [23].

One of the promising areas is the use of mixed saliva to predict the response to NACT. Thus, it was shown that in patients with a good response to NACT for oral squamous cell carcinoma, the levels of salivary microRNA-21 and -155 were lower, and the level of microRNA-375 was higher than in patients with resistant tumors [24]. The involvement of specific salivary microRNAs in rectal adenocarcinoma tumor regression was confirmed [25]. The species diversity of the microbiome and the level of salivary esterases in patients without a treatment response were lower than in patients with a treatment response for oral cancer [26]. The results of metagenomics also showed that low overall survival of patients is associated with a higher relative abundance of *Stenophotromonas*, *Staphylococcus*, *Centipeda*, *Selenomonas*, *Alloscordovia* and *Acitenobacter* [27]. Although there have been some attempts to analyze the prognostic significance of salivary parameters in NACT, no studies have been conducted for breast cancer to date.

Breast cancer has a systemic effect on the body, a key feature of which is that the malignant tumor can induce profound morphofunctional changes in tissues and organs not directly affected by the tumor, and later, throughout the body [28]. These changes will also be reflected in changes in the composition of saliva [29,30]. Previously, we formulated the hypothesis that all components of cancer cell metabolism, including changes in energy metabolism, biosynthesis, and regulation of metabolic processes, will affect changes in the metabolic composition of saliva in breast cancer [31]. It was proposed to pay special attention to the use of glutamine and fatty acids as additional or alternative energy substrates for glucose (amino acids, proteins), a high level of lipid biosynthesis (lipoproteins), increased production of reactive oxygen species and nitrogen, and, consequently, constant activation of antioxidant defense mechanisms (enzymes, DNA damage), as well as cancer markers [31,32,33,34]. Since tumor metabolism is accompanied by specific biochemical changes, it can also be used for diagnosis, prognostic assessment, and monitoring of treatment response [35,36]. Despite modern advances in medicine, predicting treatment response and patient prognosis remains challenging [37]. In this study, we examine the potential applicability of saliva metabolic parameters to assess response to neoadjuvant systemic therapy compared with traditional pathomorphological examination.

The aim of the work was a comprehensive analysis of clinical, pathological, molecular biological and salivary characteristics in assessing the response to NACT in breast cancer.

## 2. Results

### 2.1. Clinical, Pathological and Molecular Biological Characteristics of Breast Cancer Depending on Pathological Response to Therapy

Table 1 systematizes data on the structure of the study group and subgroups depending on pathological response to therapy.

**Figure 1:**
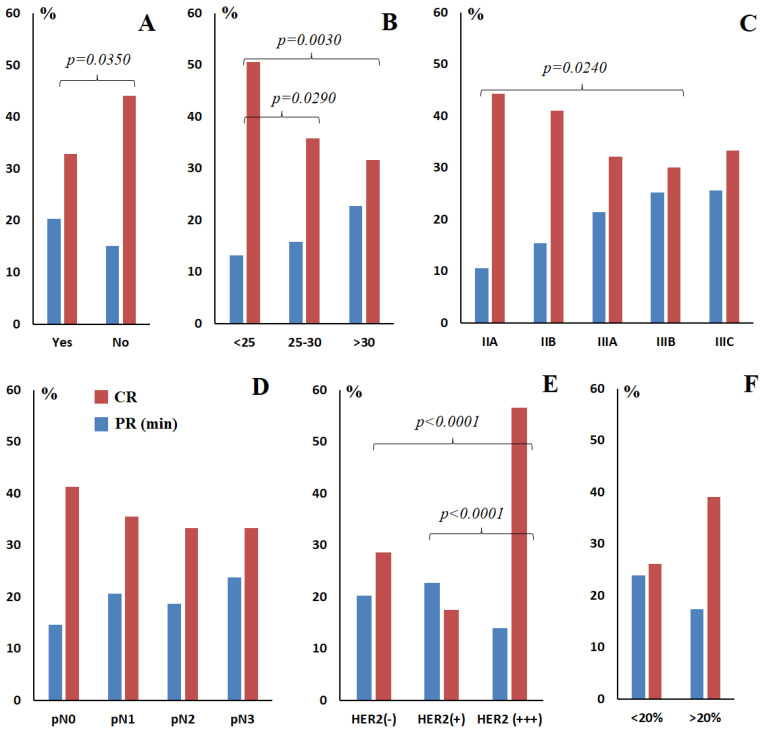

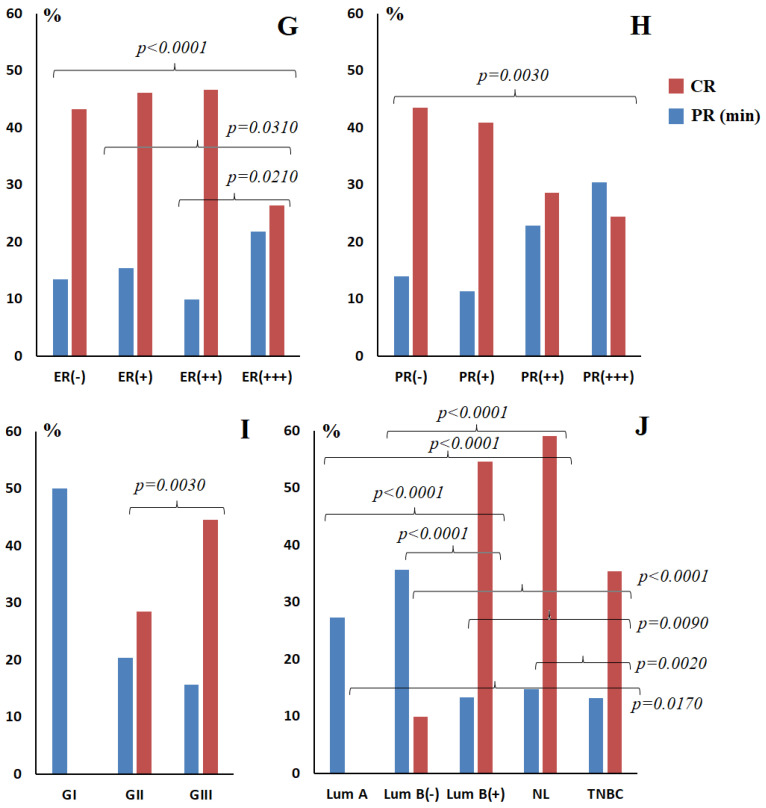
Percentage of patients with complete response to therapy (red) and minimal response to therapy (blue) depending on the clinical, pathological and molecular biological characteristics of breast cancer: (**A**)—menopause status (Yes/No); (**B**)—BMI; (**C**)—stage; (**D**)—lymph node involvement status; (**E**)—HER2 status; (**F**)—Ki-67 expression; (**G**)—estrogen receptor (ER) expression; (**H**)—progesterone receptor (PR) expression; (**I**)—degree of differentiation (GI—high, GII—moderate, GIII—poor); (**J**)—breast cancer phenotype. Lum A—luminal A, Lum B(−)—luminal B HER2-negative, Lum B(+)—luminal B HER2-positive, NL—non-luminal HER2-positive, TNBC—triple-negative breast cancer, BMI—body mass index, CR—complete response, PR (min)—minimal partial response. (−)—lack of expression; (+)—weak expression, a small number of receptors in some cells; (++)—moderate expression, a significant number of receptors in cells; (+++)—high expression of the corresponding receptor (ER, PR, HER2).

Statistically significant differences in response to therapy were shown between subgroups depending on menopausal status, BMI, ER status, HER2 status, degree of differentiation and phenotype of breast cancer (Table 1, Figure 1). Thus, the proportion of patients with a complete response to therapy was statistically significantly lower after menopause (OR = 0.621; 95% CI 0.393–0.979; *p* = 0.0350) (Figure 1A), but was higher at BMI < 25 both compared to BMI = 25–30 (OR = 1.922; 95% CI 1.044–3.563; *p* = 0.0290) and compared to BMI ˃ 30 (OR = 2.318; 95% CI 1.293–4.181; *p* = 0.0030) (Figure 1B). The relationship between the response to therapy and the stage has not been shown, with the exception of the pair IIA vs. IIIB (OR = 1.929; 95% CI 1.061–3.545; *p* = 0.0240) (Figure 1C), as well as the degree of lymph node damage (Figure 1D) and the index of proliferative activity (Figure 1F).

The proportion of patients with a complete response to therapy in the HER2(+) (HER2-low) subgroup was the lowest (17.5% vs. 28.6% for HER2(−), 17.5% vs. 56.7% for HER2(+++)) (Figure 1E). Compared with the HER2(+++) subgroup, the chance of a complete response to therapy was significantly reduced for the HER2(−) subgroup (OR = 0.308; 95% CI 0.185–0.507; *p* = 8.28 × 10^−7^) and for the HER2(+) subgroup (OR = 0.165; 95% CI 0.068–0.364; *p* = 5.38 × 10^−7^).

When assessing the impact of estrogen receptor expression status on the complete pathological response to therapy, it was shown that a high level of ER(+++) expression has an unfavorable prognosis (Figure 1G). In particular, an increase in risk was shown compared to ER(−) (OR = 2.384; 95% CI 1.399–4.135; *p* = 9.13 × 10^−4^), ER(+) (OR = 2.669; 95% CI 1.005–7.038; *p* = 0.0310) and ER(++) (OR = 2.725; 95% CI 1.090–6.799; *p* = 0.0230) statuses. For progesterone receptor expression, an increased risk was shown only for PR(+++) compared to PR(−) (OR = 2.380; 95% CI 1.302–4.487; *p* = 0.0030) (Figure 1H). A decrease in the proportion of positive responses to therapy in GII was also shown (OR = 0.495; 95% CI 0.302–0.803; *p* = 0.0030) (Figure 1I).

The lowest rate of treatment-related pathology was observed in luminal B HER2-negative breast cancer, whereas the highest rate of treatment-related pathology was observed in HER2-positive breast cancer subtypes (luminal B and non-luminal) (Figure 1J). The luminal A subtype had a significantly lower complete response rate compared with TNBC (OR = 0.00; 95% CI 0.00–0.766; *p* = 0.0170), non-luminal (OR = 0.00; 95% CI 0.00–0.315; *p* = 3.98 × 10^−4^), and luminal B HER2-positive (OR = 0.00; 95% CI 0.00–0.366; *p* = 5.44 × 10^−4^). Similarly, for the luminal B HER2-negative subtype, the proportion of complete responses to therapy was statistically significantly lower compared to TNBC (OR = 0.204; 95% CI 0.073–0.490; *p* = 6.47 × 10^−5^), non-luminal (OR = 0.079; 95% CI 0.026–0.210; *p* = 2.02 × 10^−9^) and luminal B HER2-positive (OR = 0.094; 95% CI 0.032–0.240; *p* = 6.41 × 10^−9^). No differences in the frequency of complete pathological response to therapy were found between HER2-positive subtypes (luminal B and non-luminal). A significant increase in the proportion of complete responses to therapy was established in non-luminal (OR = 2.613; 95% CI 1.359–5.092; *p* = 0.0020) and luminal B HER2-positive breast cancer (OR = 2.191; 95% CI 1.197–4.040; *p* = 0.0090) compared to TNBC.

To identify predictors associated with achieving a complete response to therapy, a multivariate logistic regression analysis was performed. The dependent variable was complete response to treatment (1 = “complete response” (Grade IV), 0 = “incomplete response” (Grades I–III)). All factors that demonstrated statistical significance in the univariate analysis were included in the model as independent variables: HER2 expression status (“+++” vs. “+/−”), estrogen and progesterone receptor expression status (“+++” vs. “++/+/−”), differentiation grade (“GIII” vs. “GI/GII”), BMI (“<25” vs. “˃25”), and menopausal status (“yes” vs. “no”). A stepwise analysis of independent variables revealed only one statistically insignificant variable (“progesterone receptor expression status,” *p* = 0.0671). The remaining variables were included in the final predictive model. The resulting logistic regression model was statistically significant (χ^2^ = 14.19, *p* < 0.0001). According to ROC analysis, the AUC was 0.756 (95% CI: 0.691–0.784); *p* < 0.0001).

### 2.2. Features of Saliva Composition Depending on Pathological Response to Therapy

**Figure 2:**
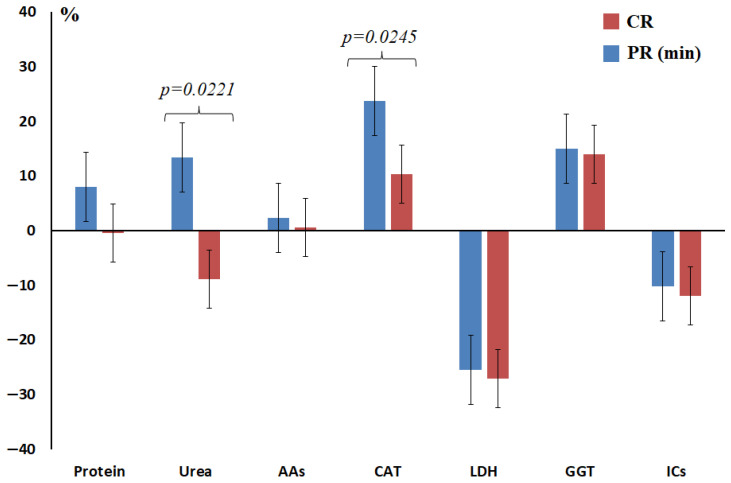
Relative concentration and activity of enzymes in saliva depending on the degree of therapeutic pathomorphosis of breast cancer compared to the healthy control (%). The relative concentration is calculated as the difference between the concentrations in the studied subgroup minus the concentration for the healthy control, divided by the concentration in the healthy control group, expressed in %. AAs—α-amino acids, CAT—catalase, LDH—lactate dehydrogenase, GGT—gamma-glutamyl transferase, ICs—imidazole compounds, CR—complete response, PR (min)—minimal partial response.

A number of biochemical parameters did not change depending on the degree of therapeutic pathomorphosis (GGT, total content of α-amino acids), while some changed insignificantly and unevenly (imidazole compounds, total protein and LDH) (Table 2). Two parameters decreased with an increase in the degree of pathomorphosis: urea concentration (−19.6% IV vs. I) and catalase activity (−10.8% IV vs. I) (Figure 2). When comparing the values of the parameters with stage I of pathomorphosis, a statistically significant decrease in the activity of LDH (*p* = 0.0002) and catalase (*p* = 0.0245) was noted, whereas for urea the decrease in concentration was statistically insignificant (Figure 2, Table 2). Thus, favorable prognostic factors include a decrease in the concentration of urea in saliva below the values in the healthy control group and a decrease in the activity of catalase (Figure 2). Moreover, a decrease in urea concentration below its content in the healthy control (7.5 mmol/L) increased the chances of response to therapy (OR = 1.921; 95% CI 1.061–4.270; *p* = 0.0342), while salivary catalase activity below 3.30 nkat/L did not show statistical significance (OR = 1.390; 95% CI 0.602–3.224; *p* = 0.3295).

**Figure 3:**
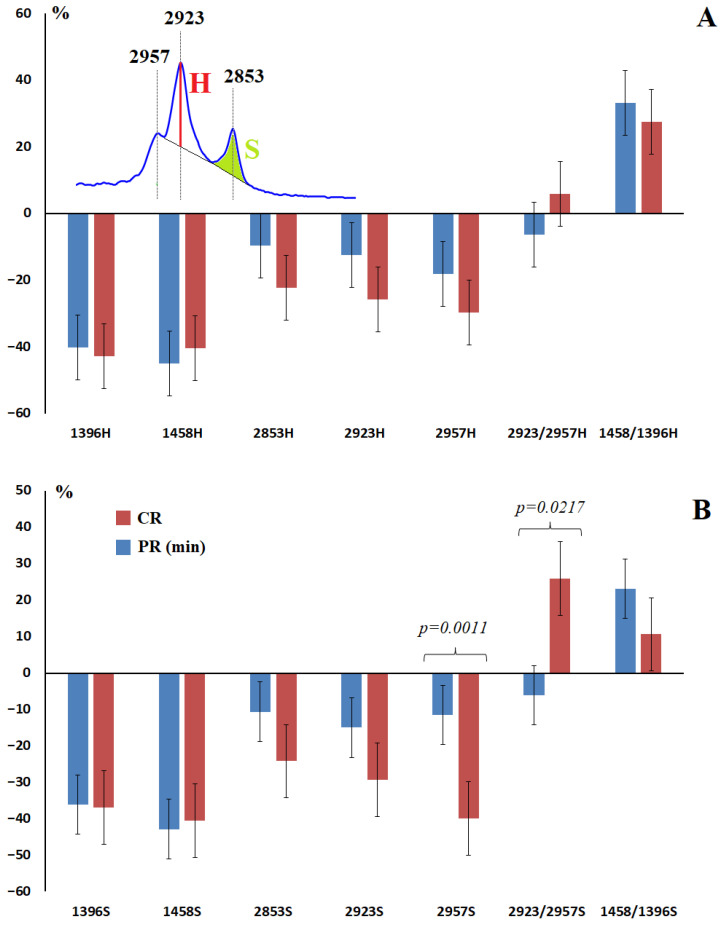
Relative intensities (**A**) and areas (**B**) of lipid absorption bands in the IR spectra of saliva depending on the degree of therapeutic pathomorphism of breast cancer compared to the healthy control (%). CR—complete response, PR (min)—minimal partial response. S—area, H—intensity.

For the absorption bands corresponding to the vibrations of methyl and methylene groups in the structure of salivary lipids (Table 3), an ambiguous pattern of change was shown depending on pathological response to therapy (Figure 3). Thus, the intensities and areas of the absorption bands at 1396 and 1458 cm^−1^ showed no patterns with pathological response to therapy (Figure 3A,B). The intensities and areas of the absorption bands at 2853, 2923 and 2957 cm^−1^ increased up to the III degree of pathomorphism, and sharply decreased at complete pathomorphism (Table 3). Two characteristics, in our opinion, can be used to predict the pathological response to therapy—the area of the absorption band at 2957 cm^−1^ and the ratio of the areas of the absorption bands at 2923/2957 cm^−1^ (Figure 3B). The choice of these characteristics was due to the presence of statistically significant differences in minimal and complete pathological response to therapy (Table 3). Thus, a decrease in the area of the absorption band at 2957 cm^−1^ and/or an increase in the ratio of the areas of the absorption bands at 2923/2957 cm^−1^ can be considered prognostically favorable signs (Figure 3B). Thus, the area of the absorption band at 2957 cm^−1^ statistically significantly decreased for Grades III (−32.3%, *p* = 0.0282) and IV (−40.0%, *p* < 0.0001) of pathomorphism. The ratio of the areas of the absorption bands at 2923/2957 cm^−1^ statistically significantly increased for Grades III (+19.4%, *p* = 0.0346) and IV (+26.0%, *p* = 0.0038). It was shown that with a decrease in the area of the absorption band at 2957 cm^−1^ (threshold value 24 as in the healthy control), the probability of a complete response to therapy was higher (OR = 3.875; 95% CI 1.160–12.70; *p* = 0.0003), whereas with an increase in the ratio of the areas of the absorption bands at 2923/2957 cm^−1^ (threshold value 12 as in the healthy control), the increase in the response to therapy was statistically insignificant (OR = 2.119; 95% CI 0.867–5.112; *p* = 0.1127).

**Figure 4:**
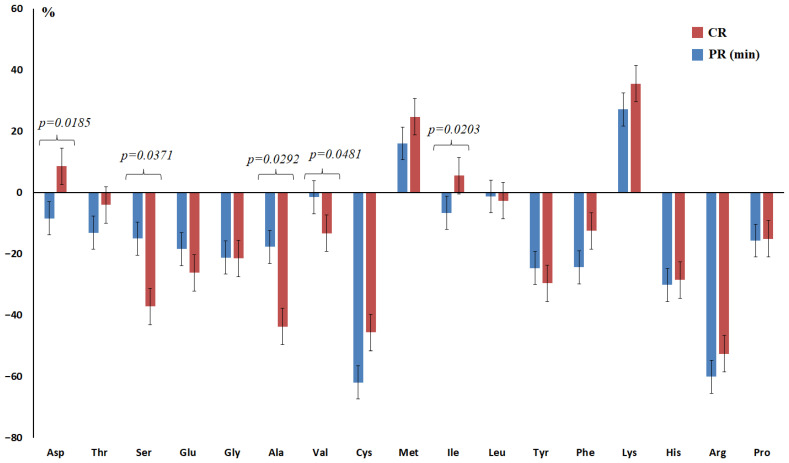
Relative salivary amino acid concentrations depending on the degree of therapeutic pathomorphism of breast cancer compared to healthy controls (%). CR—complete response, PR (min)—minimal partial response.

The concentration of amino acids in saliva varied ambiguously depending on pathological response to therapy (Table 4). Statistically significant differences between the amino acid levels in partial and complete responses to therapy were shown for Asp (*p* = 0.0185), Ser (*p* = 0.0371), Ala (*p* = 0.0292), Val (*p* = 0.0481), and Ile (*p* = 0.0203) (Table 4, Figure 4). Of all the amino acids, those with concentrations lower than those in the healthy control at Grades I and II, but higher at Grades III and IV pathomorphism, were of interest. These include Asp and Ile (Figure 4). Thus, an increase in the concentration of Asp in saliva above 1.26 nmol/L and Ile above 10.2 nmol/L (as in the healthy control) can be considered a favorable prognostic sign (Figure 4). However, the calculation of the risk ratio did not show statistical significance of the identified patterns (OR = 1.810; 95% CI 0.519–6.284; *p* = 0.1943 and OR = 1.591; 95% CI 0.620–4.067; *p* = 0.3213, respectively).

**Figure 5:**
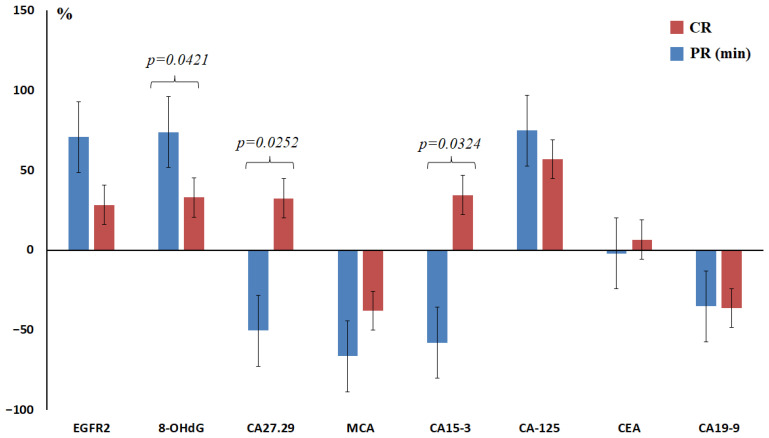
Relative concentrations of tumor markers in saliva depending on the degree of therapeutic pathomorphism of breast cancer compared to healthy controls (%). CR—complete response, PR (min)—minimal partial response.

An increase in the concentration of 8-OHdG in saliva was established, which was most pronounced at Grades I and II of therapeutic pathomorphosis (+73.8%, +112.3%, +51.5% and +32.9%) (Figure 5). Tumor markers CA27.29 (−50.3%, +11.2%, +5.1% and +32.3%) and CA15-3 (−58.0%, −60.6%, −12.6% and +34.4% for Grades I, II, III and IV, respectively) can also be distinguished; their concentration in saliva increases with an increase in the degree of tumor pathomorphosis (Figure 5). An increase in the concentration of tumor markers CA27.29 and CA15-3 and a decrease in the concentration of 8-OHdG in saliva can be considered prognostically favorable (Figure 5). Additionally, the CA15-3/8-OHdG × 100 ratio was calculated and shown to increase sharply with complete therapeutic pathomorphism of breast cancer (Table 5). The increases in this ratio compared to Grade I pathomorphism were +194.2%, +501.8%, and +736.3% for Grades II, III, and IV, respectively. Thus, the CA15-3/8-OHdG × 100 ratio is potentially informative for predicting the response to NACT in breast cancer.

For practical application, the most interesting is the use of tumor markers, the values of which change in different directions with a partial and complete response to therapy. These markers include CA27.29 (cutoff value of 3 U/L, as in the healthy control) and CA15-3 (cutoff value of 39 U/L, as in the healthy control). An increase in concentrations was prognostically favorable, while the probability of a complete response to therapy increased for both CA27.29 (OR = 2.138; 95% CI 1.021–7.273; *p* = 0.0343) and CA15-3 (OR = 3.896; 95% CI 1.062–14.07; *p* = 0.0072). With a simultaneous increase in both tumor markers, the probability of a complete response to therapy increased (OR = 4.288; 95% CI 1.056–17.09; *p* = 0.0013).

To identify predictors associated with achieving a complete response to therapy, a multivariate logistic regression analysis was performed using salivary parameters. The dependent variable was a complete response to treatment (1 = “complete response” (Grade IV), 0 = “incomplete response” (Grades I–III)). All factors that demonstrated statistical significance in the univariate analysis were included in the model as independent variables: urea concentration (“<7.5 mmol/L” vs. “˃7.5 mmol/L”), CA27.29 (“<3 U/mL” vs. “˃3 U/mL”), CA15-3 (“<39 U/mL” vs. “˃39 U/mL”), the area of the absorption band at 2957 cm^−1^ (“<24 c.u.” vs. “˃24 c.u.”), and the ratio of the areas of the absorption bands at 2923/2957 cm^−1^ (“<12 c.u.” vs. “˃12 c.u.”). The threshold values corresponded to the values of these indicators in the control group. When conducting the analysis by stepwise inclusion of independent variables, two statistically significant variables were identified (“CA15-3”, *p* = 0.0233; “2957 cm^−1^”, *p* = 0.0261). The constructed logistic regression model turned out to be statistically significant (χ^2^ = 8.01, *p* = 0.0012). According to the ROC analysis, the AUC was 0.682 (95% CI: 0.641–0.734); *p* = 0.0171).

When combining clinical, molecular biological and salivary parameters into a single model, the expression status of HER2, estrogen receptors, differentiation degree, BMI, menopause status, and CA15-3 concentration retained statistical significance. According to the ROC analysis, the AUC was 0.789 (95% CI: 0.737–0.842); *p* = 0.0001).

## 3. Discussion

Currently, clinicopathological and molecular biological characteristics are used as the main prognostic indicators of response to NACT in breast cancer. A nomogram has been developed that includes age, stage, Ki-67 index, HER2 status, and the expression status of estrogen and progesterone receptors before NACT, demonstrating good discrimination at the level of 76–79% [38]. It is known that determination of the molecular subtype helps in choosing a treatment protocol in patients with HER2-overexpressing breast cancer and TNBC, which have a better clinicopathological response to NACT than luminal subtypes [39]. For patients with luminal HER2-negative breast cancer stage IIA-IIIC, significant factors influencing the pathological response were the grade of tumor malignancy (G2/3 vs. G1), menopausal status, and intrinsic subtype (luminal B vs. A) [40]. In a multivariate analysis, a Ki-67 index > 35% and HER-2 positivity were the only independent predictors of complete pathological response [41]. NACT outcomes depend on the breast cancer subtype, and therefore, identifying sensitive and specific predictors of treatment response for each phenotype will enable early detection of chemoresistance and residual disease, reducing the risk of treatment failure and increasing overall survival.

Our results do not contradict those described in the literature; however, we also demonstrated the influence of the BMI factor on the effectiveness of NACT. In particular, patients with a BMI < 25 had a higher proportion of partial and complete responses to therapy, while with a BMI > 30, on the contrary, Grades I and II of treatment pathomorphosis were observed more often. An important observation is the lower proportion of complete responses to NACT for HER2-low breast cancer. Thus, HER2-low tumors have specific biological characteristics, and therefore may respond differently to NACT and have a different prognosis [42]. This is consistent with the literature data that tumors with a zero HER2 level had a significantly higher rate of complete remission than tumors with a low HER2 level [43], and the local response to treatment in the HER2-low group was worse [44]. HER2-low patients had a significantly lower rate of complete pathological response compared with HER2-null in the overall population (odds ratio [OR] = 0.68, *p* < 0.001) and in the hormone receptor-positive subgroup (OR = 0.73, *p* = 0.009), but not in the hormone receptor-negative subgroup (OR = 0.99, *p* = 0.755) [45].

The search for prognostically important metabolites in biological fluids and breast tissues may be no less useful than the assessment of the clinical, pathological, and molecular biological characteristics of breast cancer. We previously demonstrated that the salivary metabolomic profile significantly correlates with breast cancer phenotype [31]. Furthermore, after breast cancer surgery, the biochemical composition of saliva was restored, which may provide significant evidence for a link between changes in the salivary metabolomic profile and breast cancer [46]. This study demonstrated for the first time that a number of salivary metabolites could be informative for assessing therapy response in breast cancer. As one of the criteria for selecting metabolites, we used the multidirectional change in concentration during minimal and complete pathological responses compared to healthy control samples. These metabolites included urea, the amino acids Asp and Ile, the absorption band at 2957 cm^−1^, the 2923/2957 cm^−1^ ratio, and the tumor markers CA27.29 and CA15-3.

We have shown that lipid metabolism parameters in saliva are potentially informative metabolites. In particular, a decrease in the area of the 2957 cm^−1^ absorption band and/or an increase in the ratio of the areas of the 2923/2957 cm^−1^ absorption bands can be considered prognostically favorable signs. The range of 3050–2800 cm^−1^ contains vibrations of methyl and methylene groups of saturated and unsaturated alkyl chains in the lipid structure. The intensity ratio of 2923/2957 cm^−1^ shows the ratio of unbranched and branched lipid molecules and fatty acids (CH_2_/CH_3_). Thus, an increase in the proportion of branched lipid molecules and fatty acids in saliva is prognostically favorable.

Dysregulation of monounsaturated, polyunsaturated, and saturated fatty acids due to de novo synthesis and hypoxia is known to be a major metabolic feature of breast tumors [47]. Cheung et al. hypothesized that peritumoral lipid composition and hypoxia may be prognostic and early markers of response in breast cancer patients undergoing NACT. Díaz et al. showed that glycocholic and glycodeoxycholic acids allowed classification of TNBC patients according to treatment response and overall survival with an area under the curve (AUC) > 0.77 [48]. Stearoyl-CoA desaturase 5 is an integral membrane protein of the endoplasmic reticulum involved in lipid metabolism, which may serve as a prognostic biomarker of pathological response to NACT and play a carcinostatic role in breast cancer [49]. Fang et al. observed significant changes in serum metabolite levels before and after NACT, with a predominant enrichment of sphingolipid and amino acid metabolism pathways [50]. The authors showed that a favorable treatment response was associated with acylcarnitine levels and essential amino acid metabolism. Talarico et al. identified four metabolites (histidine, lactate, serine, and taurine) in serum that were significantly associated with progression-free survival [23]. A metabolite-related survival (MRSS) score was developed that allows stratification of patients into low- and high-risk groups for relapse, independent of classical prognostic factors. In patients in the high-risk group, the hazard ratio was 3.42, adjusted for disease stage and age. Silva et al. developed a panel of 19 compounds to predict response to NACT with high sensitivity (93.3%), specificity (100%), and accuracy (94.7%) [51]. The compounds represented in the model were lipids (glycerophospholipids and fatty acyl groups), amino acids, and bile acids and their derivatives. Jingjing et al. showed that in gastrointestinal tumors, most of the metabolites with elevated levels in saliva belonged to the category of amino acids and their derivatives [52]. These metabolites play an important role in intestinal barrier restoration, inflammation, and gastrointestinal tumor development [53,54]. For example, butyric acid can enhance the integrity of tight junctions in intestinal epithelial cells, maintaining the barrier function of the mucosa. This process is impaired in inflammatory bowel diseases due to a decrease in the number of butyric acid-producing bacteria [55,56]. Changes in the oral microbiota and its metabolites may be associated with various systemic diseases and tumors [57,58]. Thus, chronic intestinal inflammation may be associated with tumor development [59,60]. Changes in the oral microbiota have been observed in patients who underwent surgery after radiation therapy and in those who underwent surgery without radiation therapy, which was associated with increased levels of various intestinal metabolites [61]. Thus, damage to the intestinal mucosal barrier may be associated with changes in salivary metabolite levels, which may help predict patient response to radiation therapy and cancer outcomes. In this study, we demonstrated that similar changes in salivary composition associated with response to NACT could be observed in breast cancer.

Elevated Glu levels can be interpreted as a sign of its enhanced metabolism in the tumor [62]. BCAAs activate protein synthesis and promote breast cancer proliferation [63]. In general, increased amino acid levels correlate with pro-inflammatory and immunological factors [64]. Metabolic pathway analysis showed that in drug-resistant cell lines, arginine and proline metabolism, glutathione metabolism, and beta-alanine metabolism were significantly impaired compared to the parental cell line [65]. Thus, the obtained data indicate the potential of using salivary amino acid analysis to characterize metabolic homeostasis and the response to NACT in breast cancer.

We have shown that an increase in the concentration of tumor markers CA27.29 and CA15-3 in saliva and a decrease in the concentration of 8-OHdG can be considered prognostically favorable. It is known that the presence of ctDNA and elevated levels of tumor markers (CEA, CA19-9, CA125, CA72-4 and CA242) were independent prognostic factors in multivariate Cox analysis (OR = 9.771, *p* = 0.045) in gastric cancer [66]. However, the prognostic role of tumor markers in breast cancer has not been shown. Other markers, such as HIF-1α, TWIST-1, ITGB-1 and Ki-67, have proven themselves well in predicting a favorable response to treatment and resistance to NACT in breast cancer [67]. Platelets, eotaxin, interferon-gamma, interleukin-10, and transforming growth factor beta-2 have been shown to be significant predictors of complete response to NACT [68]. Low serum 8-OHdG levels were statistically significantly correlated with lymphatic vessel invasion and lymph node involvement [69], but decreased 8-OHdG levels in saliva were an indicator of treatment response. Low 8-OHdG levels in saliva may indicate impaired repair of oxidatively damaged DNA or enhanced antioxidant defense [70]. Interestingly, an increase in the concentration of the tumor markers CA15-3 and CA27.29 in saliva was a favorable prognostic factor in assessing the response to therapy. We previously demonstrated that, in general, concentrations of these markers decreased in breast cancer patients compared to healthy controls [71]. Thus, an increase in the concentrations of CA27.29 and CA15-3 in saliva corresponds to their concentrations approaching those of healthy controls and, therefore, can be considered a favorable prognostic indicator.

The prognostic significance of salivary biochemical parameters was expressed to a lesser extent; however, favorable prognostic factors include a decrease in salivary urea concentration below the values of the healthy control group, and a decrease in catalase and LDH activities. Multivariate analysis showed that the lactate dehydrogenase-to-albumin ratio (LAR) ≥ 46.27 and a positive HER2 test result are independent prognostic factors for complete clinical response (OR = 2.851, *p* = 0.025, and OR = 3.431, *p* = 0.026, respectively) [72]. Plasma LDH and its fluctuations during first-line therapy predict progression-free survival and overall survival in metastatic breast cancer [73]. It has been suggested that changes in the expression of antioxidant enzymes (in particular catalase) may be a mechanism for cancer cell resistance to chemotherapeutic drugs whose action is based on oxidation-reduction processes [74]. A prognostically favorable decrease in the concentration of urea in saliva may be associated with a decrease in the concentration of a number of amino acids during a complete pathological response to NACT.

At this stage, the mechanism underlying the observed changes in metabolites, including urea, the amino acids Asp and Ile, the absorption band at 2957 cm^−1^, the 2923/2957 cm^−1^ ratio, and the tumor markers CA27.29 and CA15-3 in saliva associated with breast cancer therapy response is not fully understood. These parameters were selected using statistical analysis, and their biological role requires further clarification, which is planned for future research. At this stage, the results are of an exploratory nature and require further validation for use in making treatment decisions.

This study’s limitations stem from the small sample size, making it impossible to analyze the impact of all parameters separately for each molecular biological subtype of breast cancer. The interpretation of salivary metabolites was performed without considering their possible bacterial origin, which in some cases complicates the attribution of metabolites to the oral metabolome or metabolic reactions of the body. The groups of parameters (amino acids, lipids, and tumor markers) were analyzed in samples of varying sizes and structures, so it was impossible to simultaneously analyze the impact of factors from different groups on the response to NACT. We plan to expand the list of parameters measured and conduct parallel measurements in a larger sample as the study continues. We also plan to develop a nomogram for a comprehensive assessment of clinical, pathological, molecular biological and salivary characteristics in breast cancer to predict treatment response.

## 4. Materials and Methods

### 4.1. Study Design

The study design included a single saliva collection and oral examination of patients strictly prior to the start of the first course of chemotherapy for breast cancer. After completion of planned neoadjuvant treatment, patients underwent surgery, after which the histological and pathological response to therapy was determined. We compared the metabolic composition of saliva, using parameters previously identified as diagnostically significant [28], with the pathological response to therapy.

The study included 361 patients with breast cancer, and 127 healthy volunteers without mammary gland pathologies. The breast cancer group included patients strictly prior to treatment with no signs of active infection or inflammatory processes in the oral cavity. All volunteers underwent an oral examination by a dentist to rule out oral diseases and periodontitis, which could affect the composition of saliva. Patients with untreated caries, acute periodontitis, or inflammatory oral diseases were excluded from the study. Healthy volunteers were recruited in the blood transfusion department and, according to routine mammography and ultrasound examinations, had no breast pathology. The volunteers were confirmed to have no clinically significant systemic diseases, as well as no history of oncological diseases.

### 4.2. Neoadjuvant Treatment Regimens

In all patients, the presence of invasive breast cancer of primary operable (stages T_1–3_N_0–1_) or locally advanced primary non-operable (stages IIIA (except T_3_N_1_M0), IIIB and IIIC) types was confirmed. All recommended courses of NACT were performed before surgery in full doses at prescribed intervals. For HER2-negative subtypes, chemotherapy was performed according to the AC regimen (doxorubicin 60 mg/m^2^ + cyclophosphamide 600 mg/m^2^ once every 3 weeks, 4 cycles) in combination with docetaxel 75 mg/m^2^ once every 3 weeks (4 cycles) or paclitaxel 80 mg/m^2^ weekly (12 injections). For HER2-positive breast cancer, chemotherapy was supplemented with targeted therapy—trastuzumab 6 mg/kg (loading dose 8 mg/kg) once every 3 weeks (4 cycles).

### 4.3. Determination of Pathological Response to Therapy

After completion of the neoadjuvant phase, all patients underwent surgery. Pathological response was assessed centrally in the pathology department of the Clinical Oncology Center using postoperative specimens. Patients were divided into four groups depending on the degree of therapeutic pathomorphosis: I (more than 50% of tumor parenchyma preserved)—66 (18.3%), II (20–50% of tumor parenchyma preserved)—83 (23.0%), III (up to 20% of tumor parenchyma preserved in the form of separate foci)—77 (21.3%), and IV (complete absence of tumor parenchyma)—135 (37.4%) patients. Grade IV was characterized by complete tumor regression (complete pathological response). The primary endpoint of the study was the achievement of a complete pathological response (ypT0/is ypN0). Binary modeling (“yes/no” response) was used to calculate the relative risk. Ordinal modeling (Grades I–IV) was used to estimate more subtle variables, which may contribute to understanding the mechanism of these changes.

### 4.4. Methods of Collection, Storage, Transportation and Pre-Processing of Saliva

Mixed saliva (oral fluid) forms in the oral cavity. Its composition differs from that of the mixture of glandular secretions, since microorganisms and their waste products, various food components, and plaque components are present in the oral fluid. In this paper, we are talking about mixed saliva (oral fluid), which is referred to as “saliva” for simplicity.

Before the first course of NACT, saliva samples were collected from all study participants. Saliva was collected on an empty stomach after a night’s sleep without any additional stimulation in sterile polypropylene centrifuge tubes with screw caps. From the moment they woke up until the saliva collection, volunteers were asked to abstain from smoking, drinking water, and taking medications. Immediately before collecting the saliva samples, volunteers rinsed their mouths with water. Saliva samples of inadequate quality, including those with signs of blood contamination, were excluded from the study. Samples were collected between 8 and 10 a.m. by spitting; the collection time was 10 min, and the volume of saliva was 3–4 mL. Immediately after collection, the tubes were refrigerated at 2–8 °C and stored for no more than 4–6 h. Upon delivery to the laboratory, the tubes were centrifuged at 10,000× *g* (CLn-16, Russia) for 10 min, the supernatant was removed, and the samples were transferred to Eppendorf tubes for further storage at −86 °C.

### 4.5. Determination of the Amino Acid Composition of Saliva by HPLC

Immediately before analysis, the samples were thawed, 1 mL of the sample was collected in a centrifuge tube, 0.3 mL of 23% trichloroacetic acid solution was added to precipitate the protein, and the tube contents were mixed well, and left for 15 min. The samples were then centrifuged at 17,000× *g* for 15 min at 4 °C (Sigma 1-15 PK, Sigma, Osterrode am Harz, Germany). The sediment was discarded, and the supernatant was filtered through a 0.45 µm syringe filter (CHROMAFIL CA-45/25, Macherey-Nagel, Düren, Germany) before analysis.

The analysis was performed on an LA8080 automatic analyzer (Hitachi, Tokyo, Japan) equipped with an 80 × 4.6 mm column with 2622 ion-exchange resin (Hitachi, Tokyo, Japan) and a pre-column for ammonium peak suppression, a post-column reactor for amino acid modification with ninhydrin, and a spectrophotometric detector. The analyzer unit operating parameters were as follows: column temperature of 20–90 °C, operating pressure of 1.6 MPa, eluent flow of 0.2 mL/min, and measuring channels with wavelengths of 440 nm and 570 nm. The analysis was performed using an adapted program that included the supply of five eluents with different compositions and pH at specific temperatures and allowed for the best separation of the amino acid mixture. For quantitative assessment, a standard amino acid mixture of known concentration for calibration (Pickering calibration standard, Pickering Laboratories, Mt-View, CA, USA) was analyzed under the same conditions immediately before the analysis of a series of samples. Identification of amino acid peaks on the chromatogram was carried out using the addition method.

### 4.6. Determination of the Salivary Lipid Spectrum by Infrared (IR) Spectroscopy

To determine salivary lipids, preliminary extraction of lipids was performed with Folch solution (chloroform:ethanol = 2:1, vol.) according to the method adapted by the author, followed by analysis of the extracts by IR spectroscopy [75]. An amount of 200 μL of the sample (saliva) was diluted with 800 μL of 0.9% NaCl; then, the samples were extracted twice with 2 mL of Folch solution. The combined organic phase was settled for 24 h, and then centrifuged (10,000× *g* for 10 min) for a more complete phase separation. The upper layer was carefully decanted and the bottom layer was selected for IR spectroscopy. Extracts with a volume of 50 μL were dried for 30 min on a substrate of zinc selenide in a thermostat at 37 °C. The infrared absorption spectra were registered using an FT-801 Fourier IR spectrometer (SIMEX, Novosibirsk, Russia) in the range of 500–4000 cm^–1^. Spectra were recorded with a scan number of 32 with a resolution of 4 cm^−1^. Three spectra were collated per sample/patient. The results were presented as an averaged (or levelled) spectrum. ZaIR 3.5 software (SIMEX, Novosibirsk, Russia) was used to carry out baseline correction and normalization of FTIR spectra. The intensity (H) and area (S) of the absorption bands at 1396 cm^−1^ (δCH_3_), 1458 cm^−1^ (δCH_2_), 2853 cm^−1^ (ν_s_CH_3_), 2923 cm^−1^ (ν_as_CH_2_) and 2957 cm^−1^ (ν_as_CH_3_) were analyzed, as well as the ratios 2923/2957 and 1458/1396 cm^−1^ (ν_s_—symmetric stretching, ν_as_—asymmetric stretching and δ—deformation vibrations). The 2923/2957 cm^−1^ ratio has been suggested as informative for differentiating marginal from malignant specimens [76].

### 4.7. Determination of the Biochemical Composition of Saliva

In all saliva samples, the following were determined: total protein by reaction with pyrogallol red (mg/L, cat. No. B-8084, Vector-Best LLC, Novosibirsk, Russia), urea by the urease–salicylate method according to Berthelot (mmol/L, cat. No. B-8074, Vector-Best LLC, Novosibirsk, Russia), total content of α-amino acids by reaction with ninhydrin (α-AAs, mmol/L), and imidazole compounds by the diazotization reaction in the presence of sulfanilic acid (ICs, mmol/L). Gamma-glutamyl transferase activity was determined by the kinetic method using L-gamma-glutamyl-3-carboxy-4-nitroanilide as a substrate according to Seitz-Persin (GGT, U/L, cat. No. B-8030, Vector-Best LLC, Novosibirsk, Russia), lactate dehydrogenase by the kinetic UV method by the rate of nicotinamide adenine dinucleotide (NADH) oxidation (LDH, U/L, cat. No. B-8321, Vector-Best LLC, Novosibirsk, Russia), and catalase by determining the rate of hydrogen peroxide utilization (nkat/L, cat. No. G4307-48T, Wuhan Servicebio Technology, Wuhan, China). All parameters were measured using the StatFax 3300 semi-automatic biochemical analyzer (Awareness Technology, Palm City, FL, USA) [46].

The validation procedure for each test system included two analytical series. Each series included the analysis of calibration standards to construct a calibration curve, as well as the required number of quality control samples with a specified concentration of the corresponding indicator. Each sample was analyzed in duplicate.

### 4.8. Determination of Saliva Tumor Markers

8-OH-deoxyguanosine (8-OhdG, pg/mL) was determined by a competitive enzyme-linked immunosorbent assay (Cloud-Clone Corp, Wuhan, China, cat. No. CEA660Ge). EGFR2 (ng/mL) was determined by the sandwich enzyme immunoassay method (Cloud-Clone Corp, Wuhan, China, cat. No. CEB867Hu). The content of tumor markers CA15-3 (catalog number K226, U/mL), CA27.29 (K227, U/mL), MCA (K228, U/mL), CEA (K224, ng/mL), CA125 (K222, U/mL), and CA19-9 (K223, U/mL) in saliva was determined by the solid-phase enzyme immunoassay method using Hema kits (Moscow, Russia) using the Thermo Fisher Multiskan FC analyzer (Waltham, MA, USA). The concentration was calculated in accordance with the manufacturer’s instructions.

Validation of the ELISA for saliva analysis included standard operating procedures for commercial kits: verification of accuracy (repeatability, intermediate precision, and reproducibility), dilution linearity, analyte recovery, and sample stability. Absolute cutoff values were obtained on a limited sample and require further validation in a multicenter, interlaboratory study.

### 4.9. Statistical Analysis

Statistical analysis was performed using Statistica 13.3 EN software (StatSoft, Tulsa, OK, USA) by a nonparametric method after testing the distribution and homogeneity of variances in groups using the Shapiro–Wilk and Bartlett tests. Continuous variables were presented as the median and interquartile range (IQR). Categorical variables were shown as frequencies and their percentage (%). When comparing more than two subgroups, a Bonferroni correction was used to adjust the *p*-value: instead of adjusting the alpha significance level, each *p*-value was multiplied by the number of tests, and the alpha significance level was left unchanged (*p* < 0.05). Fisher’s exact test was used to check the significance of the relationship between categorical variables.

## 5. Conclusions

It was found that the proportion of patients with a complete response to therapy was statistically significantly lower after menopause (OR = 0.621; 95% CI 0.393–0.979; *p* = 0.0350), as well as with HER2-negative breast cancer status (OR = 0.308; 95% CI 0.185–0.507; *p* = 8.28 × 10^−7^) and moderate tumor differentiation grade GII (OR = 0.495; 95% CI 0.302–0.803; *p* = 0.0030). Prognostically unfavorable were high levels of expression of estrogen receptors (OR = 2.384; 95% CI 1.399–4.135; *p* = 9.13 × 10^−4^) and progesterone (OR = 2.380; 95% CI 1.302–4.487; *p* = 0.0030), as well as an increase in BMI to 25–30 (OR = 1.922; 95% CI 1.044–3.563; *p* = 0.0290) and more than 30 (OR = 2.318; 95% CI 1.293–4.181; *p* = 0.0030). An important result was that the lowest proportion of patients with a complete response to therapy was in the HER2-low subgroup (OR = 0.165; 95% CI 0.068–0.364; *p* = 5.38 × 10^−7^). It should be noted that the minimal degree of treatment pathomorphism was characteristic of luminal B HER2-negative breast cancer, whereas the maximum degree of pathomorphism was noted for HER2-positive breast cancer subtypes (luminal B and non-luminal).

The criterion for selecting informative salivary metabolites was a multidirectional change with minimal and complete pathological response to therapy compared to healthy controls. Thus, prognostically favorable signs were a decrease in the concentration of urea below 7.5 mmol/L (OR = 1.921; 95% CI 1.061–4.270; *p* = 0.0342), a decrease in the area of the absorption band at 2957 cm^−1^ (threshold value 24) (OR = 3.875; 95% CI 1.160–12.70; *p* = 0.0003), and an increase in the concentration of CA 27.29 above 3 U/L (OR = 2.138; 95% CI 1.021–7.273; *p* = 0.0343) and CA-15-3 above 39 U/L (OR = 3.896; 95% CI 1.062–14.07; *p* = 0.0072). With a simultaneous increase in both CA27.29 and CA15-3, the probability of a complete response to therapy increased (OR = 4.288; 95% CI 1.056–17.09; *p* = 0.0013).

Multivariate analysis showed that an independent prognostic indicator, along with the expression status of HER2, estrogen receptors, differentiation degree, BMI, and menopause status, was the concentration of CA15-3 in saliva (AUC = 0.789, 95% CI: 0.737–0.842, *p* = 0.0001).

Undoubtedly, the use of salivary biomarkers alone to predict response to NACT requires verification and full-scale clinical trials. Nevertheless, at this stage, these metabolites can be used to refine the prognosis and, in the future, to develop nomograms for comprehensive prognosis assessment.

## Figures and Tables

**Table 1:** Description of the study group depending on pathological response to therapy.

Characteristics		All Patients	The Degree of Therapeutic Pathomorphosis				*p*-Value
			I	II	III	IV	
**Number of patients**		361	66	83	77	135	
**Age, years**		54.7 ± 12.6	56.1 ± 12.8	55.9 ± 11.8	55.8 ± 13.1	52.5 ± 12.6	0.8571
**Menopause**	Yes	216 (59.8%)	44 (66.7%)	55 (66.3%)	46 (59.7%)	71 (52.6%)	0.0350 *
	No	145 (40.2%)	22 (33.3%)	28 (33.7%)	31 (40.3%)	64 (47.4%)	
**BMI**	<25	83 (23.0%)	11 (16.7%)	14 (16.9%)	16 (20.8%)	42 (31.1%)	0.0290 *
	25–30	120 (33.2%)	19 (28.8%)	27 (32.5%)	31 (40.3%)	43 (31.9%)	
	>30	158 (43.8%)	36 (54.5%)	42 (50.6%)	30 (39.0%)	50 (37.0%)	
**Stage**	IIA	113 (31.3%)	12 (18.2%)	26 (31.3%)	25 (32.5%)	50 (37.0%)	0.4702
	IIB	78 (21.6%)	12 (18.2%)	14 (16.9%)	20 (26.0%)	32 (23.7%)	
	IIIA	28 (7.8%)	6 (9.1%)	9 (10.8%)	4 (5.2%)	9 (6.7%)	
	IIIB	103 (28.5%)	26 (39.4%)	27 (32.5%)	19 (24.7%)	31 (23.0%)	
	IIIC	39 (10.8%)	10 (15.2%)	7 (8.4%)	9 (11.7%)	13 (9.6%)	
**Lymph node status**	pN_0_	150 (41.6%)	22 (33.3%)	34 (41.0%)	32 (41.6%)	62 (45.9%)	0.3976
	pN_1_	121 (33.5%)	25 (37.9%)	26 (31.3%)	27 (35.1%)	43 (31.9%)	
	pN_2_	48 (13.3%)	9 (13.6%)	15 (18.1%)	8 (10.4%)	16 (11.9%)	
	pN_3_	42 (11.6%)	10 (15.2%)	8 (9.6%)	10 (13.0%)	14 (10.4%)	
**HER2 status**	(−)	168 (46.5%)	34 (51.5%)	47 (56.6%)	39 (50.6%)	48 (35.6%)	<0.0001 *
	(+)	57 (15.8%)	13 (19.7%)	18 (21.7%)	16 (20.8%)	10 (7.4%)	
	(+++)	136 (37.7%)	19 (28.8%)	18 (21.7%)	22 (28.6%)	77 (57.0%)	
**ER status**	(−)	185 (51.2%)	25 (37.9%)	39 (47.0%)	41 (53.2%)	80 (59.3%)	0.0310 *
	(+)	26 (7.2%)	4 (6.1%)	7 (8.4%)	3 (3.9%)	12 (8.9%)	
	(++)	30 (8.3%)	3 (4.5%)	5 (6.0%)	8 (10.4%)	14 (10.4%)	
	(+++)	110 (30.5%)	24 (51.5%)	32 (38.6%)	25 (32.5%)	29 (21.5%)	
**PR status**	(−)	200 (55.4%)	28 (42.4%)	45 (54.2%)	40 (51.9%)	87 (64.4%)	0.0680
	(+)	44 (12.2%)	5 (7.6%)	9 (10.8%)	12 (15.6%)	18 (13.3%)	
	(++)	35 (9.7%)	8 (12.1%)	9 (10.8%)	8 (10.4%)	10 (7.4%)	
	(+++)	82 (22.7%)	25 (37.9%)	20 (24.1%)	17 (22.1%)	20 (14.8%)	
**Ki-67**	<20%	46 (12.7%)	11 (16.7%)	14 (16.9%)	9 (11.7%)	12 (8.9%)	0.5360
	>20%	315 (87.3%)	55 (83.3%)	69 (83.1%)	68 (88.3%)	123 (91.1%)	
**G**	I	4 (1.1%)	2 (3.0%)	1 (1.2%)	0 (0%)	1 (0.7%)	0.0030 *
	II	137 (38.0%)	28 (42.4%)	36 (43.4%)	34 (44.2%)	39 (28.9%)	
	III	204 (56.5%)	32 (48.5%)	41 (49.4%)	40 (51.9%)	91 (67.4%)	
	X	16 (4.4%)	4 (6.1%)	5 (6.0%)	3 (3.9%)	4 (3.0%)	
**Phenotype**	Lum A	11 (3.0%)	3 (4.5%)	7 (8.4%)	1 (1.3%)	0 (0%)	<0.0001 *
	Lum B(−)	70 (19.4%)	25 (37.9%)	21 (25.3%)	17 (22.1%)	7 (5.2%)	
	Lum B(+)	75 (20.8%)	10 (15.2%)	9 (10.8%)	15 (19.5%)	41 (30.4%)	
	NL	61 (16.9%)	9 (13.6%)	9 (10.8%)	7 (9.1%)	36 (26.7%)	
	TNBC	144 (39.9%)	19 (28.8%)	37 (44.6%)	37 (48.1%)	51 (37.8%)	

The percentage of the total number of patients in the corresponding subgroup is given in parentheses. Lum A—luminal A, Lum B(−)—luminal B HER2-negative, Lum B(+)—luminal B HER2-positive, NL—non-luminal HER2-positive, TNBC—triple-negative breast cancer, BMI—body mass index. *—Differences between subgroups are statistically significant according to Fisher’s exact test, *p* < 0.05. (−)—lack of expression; (+)—weak expression, a small number of receptors in some cells; (++)—moderate expression, a significant number of receptors in cells; (+++)—high expression of the corresponding receptor (ER, PR, HER2).

**Table 2:** Salivary biochemical composition depending on pathological response to therapy.

Indicators	The Degree of Therapeutic Pathomorphosis			
	I, n = 37	II, n = 38	III, n = 40	IV, n = 69
**Protein, mg/L**	0.71 [0.47; 0.92]	0.69 [0.42; 0.94]	0.63 [0.45; 0.79]	0.66 [0.47; 0.81]
**Urea, mmol/L**	8.47 [4.18; 10.41]	7.09 [5.14; 9.55]	7.03 [5.26; 10.81]	6.81 [4.04; 9.10]
	*p*_I–IV_ = 0.0221	*-*	*-*	*p*_I–IV_ = 0.0221
**α-AAs, mmol/L**	3.94 [3.77; 4.21]	3.95 [3.71; 4.45]	3.93 [3.62; 4.17]	3.88 [3.69; 4.10]
**Catalase, nkat/mL**	3.70 [2.43; 5.28]	3.98 [2.63; 6.00]	3.49 [2.58; 4.92]	3.30 [2.43; 4.94]
	*p*_I–IV_ = 0.0245	*p*_II–IV_ = 0.0178	-	*p*_I–IV_ = 0.0245, *p*_II–IV_ = 0.0178
**LDH, U/L**	952.6 [601.7; 1430.0]	1110.5 [648.1; 1643.5]	1035.0 [625.6; 1707.0]	930.9 [671.2; 1207.0]
	-	*p*_II–IV_ = 0.0002	-	*p*_II–IV_ = 0.0002
**GGT, U/L**	22.2 [16.7; 24.0]	22.1 [18.3; 25.9]	22.3 [18.1; 25.3]	22.0 [18.8; 24.8]
**ICs, mmol/L**	0.296 [0.130; 0.389]	0.287 [0.117; 0.412]	0.249 [0.130; 0.424]	0.290 [0.166; 0.462]

*p*-values are given for comparison of the corresponding degrees of therapeutic pathomorphosis (I, II, III, and IV), *p* < 0.05.

**Table 3:** Characteristics of absorption bands in the IR spectra of salivary lipid extracts depending on pathological response to therapy.

Absorption Bands, cm^−1^		The Degree of Therapeutic Pathomorphosis			
		I, n = 37	II, n = 38	III, n = 40	IV, n = 69
**1396**	H	0.71 [0.53; 1.09]	0.61 [0.47; 0.73]	0.75 [0.59; 0.81]	0.68 [0.53; 1.07]
	S	4.07 [2.96; 6.66]	3.44 [2.78; 3.94]	4.21 [3.41; 5.95]	4.01 [3.11; 6.07]
**1458**	H	1.85 [1.61; 2.27]	2.24 [1.76; 2.54]	2.06 [1.83; 3.02]	2.01 [1.64; 2.70]
	S	7.83 [5.96; 9.74]	9.69 [7.04; 10.40]	9.21 [7.63; 12.70]	8.15 [6.68; 11.30]
**2853**	H	6.39 [4.44; 7.19]	6.96 [4.26; 11.00]	7.75 [5.33; 9.51]	5.49 [4.24; 8.33]
	S	113.5 [77.4; 132.0]	127.0 [71.3; 217.0]	139.0 [92.7; 177.0]	96.3 [72.9; 157.0]
**2923**	H	10.75 [7.68; 11.90]	11.30 [6.86; 20.80]	12.50 [9.10; 15.60]	9.13 [6.82; 13.30]
	S	261.0 [170.0; 300.0]	279.0 [162.0; 554.0]	317.0 [219.0; 377.0]	217.0 [161.0; 331.0]
**2957**	H	2.07 [1.80; 2.46]	2.28 [1.70; 4.55]	2.10 [1.55; 3.08]	1.78 [1.54; 2.39]
	S	21.45 [14.60; 26.00]	22.25 [17.90; 67.00]	16.40 [10.00; 32.80]	14.55 [10.90; 19.40]
		*p*_I–IV_ = 0.0011	-	-	*p*_I–IV_ = 0.0011
**2923/2957**	H	4.42 [3.91; 5.51]	4.46 [3.52; 5.45]	4.75 [4.35; 6.45]	4.99 [4.47; 6.31]
	S	11.19 [6.54; 18.83]	9.11 [6.36; 10.69]	14.22 [8.71; 21.63]	15.00 [10.81; 22.37]
		*p*_I–IV_ = 0.0217	*p*_II–IV_ = 0.0013	-	*p*_I–IV_ = 0.0217, *p*_II–IV_ = 0.0013
**1458/1396**	H	3.22 [2.30; 3.42]	3.68 [3.15; 4.72]	3.04 [2.68; 3.61]	3.08 [2.56; 3.54]
	S	2.38 [1.92; 2.69]	2.93 [2.30; 3.69]	2.28 [2.05; 2.67]	2.13 [1.98; 2.62]

*p*-values are given for comparison of the corresponding degrees of therapeutic pathomorphosis (I, II, III, and IV), *p* < 0.05. S—area, H—intensity.

**Table 4:** Salivary amino acid compositions depending on pathological response to therapy. * *p* < 0.05.

AAs, nmol/L	The Degree of Therapeutic Pathomorphosis		*p*-Value
	I–III, n = 36	IV, n = 35	
**Aspartic acid (Asp)**	1.51 [0.91; 2.54]	1.27 [0.81; 1.79]	0.0185 *
**Threonine (Thr)**	1.69 [1.33; 2.63]	1.66 [1.27; 2.08]	0.2547
**Serine (Ser)**	5.35 [3.02; 8.35]	3.35 [2.11; 8.55]	0.0371 *
**Glutamic acid (Glu)**	17.2 [8.6; 27.0]	11.1 [4.8; 25.9]	0.4420
**Glycine (Gly)**	136.7 [101.6; 230.5]	111.0 [74.9; 173.0]	0.7313
**Alanine (Ala)**	56.5 [31.0; 90.4]	29.3 [19.7; 56.6]	0.0292 *
**Valine (Val)**	20.8 [12.8; 38.6]	16.4 [7.3; 27.3]	0.0481 *
**Cysteine (Cys)**	1.01 [0.46; 6.79]	0.59 [0.36; 1.01]	0.1879
**Methionine (Met)**	4.96 [2.76; 7.12]	4.51 [3.71; 6.37]	0.1367
**Isoleucine (Ile)**	10.3 [8.2; 24.5]	9.5 [6.1; 14.0]	0.0203 *
**Leucine (Leu)**	20.7 [13.0; 42.2]	15.8 [10.0; 26.2]	0.1070
**Tyrosine (Tyr)**	50.6 [34.9; 61.9]	43.5 [23.9; 56.4]	0.5493
**Phenylalanine (Phe)**	40.8 [27.7; 68.4]	41.6 [28.3; 53.9]	0.9489
**Lysine (Lys)**	45.8 [26.8; 69.6]	41.7 [21.8; 57.3]	0.0919
**Histidine (His)**	19.6 [14.0; 26.0]	16.3 [11.1; 26.1]	0.5957
**Arginine (Arg)**	10.1 [6.53; 22.4]	11.1 [4.94; 21.3]	0.4154
**Proline (Pro)**	72.2 [44.3; 127.6]	57.5 [35.8; 89.9]	0.2235

**Table 5:** Salivary tumor marker concentrations depending on pathological response to therapy.

Tumor Markers	The Degree of Therapeutic Pathomorphosis			
	I, n = 37	II, n = 38	III, n = 40	IV, n = 69
**EGFR2, ng/mL**	1.03 [0.560; 1.12]	0.560 [0.389; 1.12]	0.474 [0.389; 0.774]	0.774 [0.603; 0.944]
**8-OHdG, pg/mL**	327.6 [114.0; 1136.6]	400.0 [163.8; 636.2]	285.5 [93.0; 549.9]	250.4 [196.4; 666.9]
	*p*_I–IV_ = 0.0421	*p*_II–IV_ = 0.0124	-	*p*_I–IV_ = 0.0421, *p*_II–IV_ = 0.0124
**CA27.29, U/mL**	1.53 [0.94; 2.18]	3.43 [0.69; 15.23]	3.24 [1.67; 5.09]	4.08 [1.33; 9.32]
	*p*_I–IV_ = 0.0252	-	-	*p*_I–IV_ = 0.0252
**MCA, U/mL**	7.08 [5.67; 19.02]	19.13 [9.53; 119.9]	12.60 [8.60; 22.59]	13.10 [6.43; 80.46]
**CA15-3, U/mL**	16.64 [7.40; 32.15]	15.62 [14.09; 29.19]	34.60 [25.11; 98.65]	53.22 [18.68; 133.0]
	*p*_I–IV_ = 0.0324	-	-	*p*_I–IV_ = 0.0324
**CA-125, U/mL**	410.2 [210.4; 448.6]	282.4 [234.3; 549.2]	395.1 [258.6; 472.2]	367.8 [229.6; 450.2]
**CEA, ng/mL**	81.6 [74.3; 95.9]	92.9 [80.6; 95.0]	83.1 [75.3; 89.0]	88.8 [73.9; 97.8]
**CA19-9, U/mL**	31.91 [11.18; 65.09]	11.27 [8.45; 27.82]	38.64 [21.64; 67.55]	31.36 [14.36; 125.3]
**CA15-3/8-OHdG × 100**	2.26 [1.09; 8.21]	6.65 [2.21; 11.1]	13.6 [5.73; 19.9]	18.9 [3.96; 43.4]
	*p*_I–IV_ = 0.0112	-	-	*p*_I–IV_ = 0.0112

## Data Availability

The original contributions presented in this study are included in the article. Further inquiries can be directed to the author.
